# Advancing Computational Analysis of Porous Materials—Modeling Three-Dimensional Gas Adsorption in Organic Gels

**DOI:** 10.1021/acs.jpcb.0c11000

**Published:** 2021-02-16

**Authors:** Elisha Martin, Martin Prostredny, Ashleigh Fletcher, Paul Mulheran

**Affiliations:** Department of Chemical & Process Engineering, University of Strathclyde, Glasgow G1 1XL, United Kingdom

## Abstract

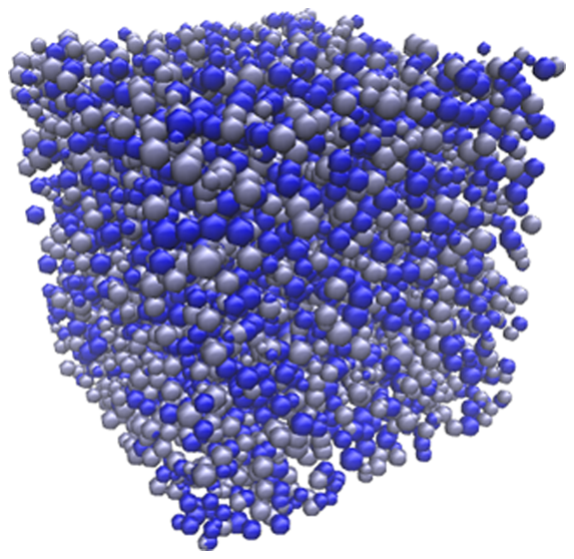

Assessing the efficacy of specific porous materials for use in various applications has been a central focus for many experimental studies over the years, with a view to altering the material properties according to the desired characteristics. The application potential for one such class of nanoporous materials—organic resorcinol-formaldehyde (RF) gels—is of particular interest, due to their attractive and adjustable properties. In this work, we simulate adsorption analysis using lattice-based mean field theory, both in individual pores and within three-dimensional porous materials generated from a kinetic Monte Carlo cluster aggregation model. We investigate the impacts of varying pore size and geometry on the adsorptive behavior, with results agreeing with those previously postulated in the literature. The adsorption analysis is carried out for porous materials simulated with varying catalyst concentrations and solids contents, allowing their structural properties to be assessed from resulting isotherms and the adsorption and desorption processes visualized using density color maps. Isotherm analysis indicated that both low catalyst concentrations and low solids contents resulted in structures with open transport pores that were larger in width, while high catalyst concentrations and solids contents resulted in structures with bottleneck pores that were narrower. We present results from both the simulated isotherms and pore size analysis distributions, in addition to results from RF gels synthesized in the lab and analyzed experimentally, with significant similarities observed between the two. Not only do the results of this comparison validate the kinetic Monte Carlo model’s ability to successfully capture the formation of RF gels under varying synthesis parameters, but they also show significant promise for the tailoring of material properties in an efficient and computationally inexpensive manner—something which would be pivotal in realizing their full application potential, and could be applied to other porous materials whose formation mechanism operates under similar principles.

## 1 Introduction

The properties of various porous materials have been investigated extensively over the years through experimental work, with a view to understanding the impacts of various synthesis parameters, in addition to optimizing such materials for use in specific applications. To date, applications for porous organic materials have ranged from gas adsorption^1^ and water treatment^2,3^ to thermal insulation^4^ and energy storage.^5^ This work focuses on resorcinol-formaldehyde (RF) gels, which are organic materials with high surface areas, low densities, and high porosities and whose exceptional structural properties can be tailored according to application requirements. Further exploring the application potential of these materials is pivotal; however, experimental studies investigating the properties of RF gels and their application performance commonly report a time-consuming synthesis process, with traditional gel formation as first described by Pekala (1989)^6^ followed by drying and analysis stages, requiring several days, occasionally weeks, to complete.^7^ Although not yet widely adopted, microwave synthesis of RF gels has proven to significantly reduce the time required for synthesis in the lab, although analysis time requirements, of course, remain the same.^8,9^ This presents an opportunity, therefore, to explore computational means of investigating the properties of porous materials such as these, allowing their characteristics to be tailored more efficiently according to application requirements.

One of the most fundamental analysis methods for porous materials is nitrogen adsorption measurements, the isotherm data from which provides crucial details on the material’s internal structure, including the size, geometry, and total volume of the pores present, as well as the adsorption behavior of the gas on the material’s surface, such as observations of monolayer or multilayer formation. Different isotherm and hysteresis types have been categorized by the International Union of Pure and Applied Chemistry (IUPAC) according to their shape, detailing the implications of these in terms of the material’s structure.^10^

Computational adsorption analysis as a means to understanding porous structures in greater detail has also been studied, providing insight into the adsorption and desorption mechanisms and allowing material properties to be determined. Multiple techniques have been employed to model the adsorption process, including classical density functional theory (DFT) calculations,^11,12^ in addition to Monte Carlo and molecular dynamics simulations, which have explored adsorption within materials such as graphite,^13^ nanoporous silica,^14^ and metal organic frameworks,^15^ producing adsorption isotherms for the simulated materials studied. Recent progress in models such as these has even led to the development of widely accessible adsorption software.^16^ Although adsorption models such as these provide valuable insights into the detailed interactions between adsorbates and adsorbents, their relevance to industrial applications is limited by their significant computational expense.^17^ Furthermore, many of the studies which utilize these methods are able to simulate adsorption within just a few structures, or within specific individual pores, as opposed to performing the analysis over a wide range of varying structures that would be valuable for tailoring materials.

In an effort to advance toward more computationally efficient adsorption analysis, more recent studies have taken a coarse-grain approach using lattice-based mean field theory (MFT), with studies focusing on understanding the adsorption mechanism within individual pores of varying sizes and geometries^18−20^ and further work extending to adsorption analysis within complex porous structures.^21,22^ This approach has also been applied to silica gels, using it predominantly as a tool to elucidate the mechanisms behind hysteresis formation.^23,24^ The work presented here builds upon this approach, applying these lattice-based MFT calculations to three-dimensional simulated porous organic gels to model adsorption analysis within materials produced across a range of synthesis parameters, allowing an extensive range of structures to be explored and analyzed, with a view to enable material tailoring.

We have previously reported our findings from a 3D model developed within our group that simulates the growth of porous organic materials, such as RF gels, from the initial monomer species through to the interconnected final cluster structure.^25,26^ This lattice-based Monte Carlo simulation has modeled the formation of RF gels across varying catalyst concentrations and solids contents—two fundamental parameters that have proven to control gel properties in experimental work—and the resulting materials were analyzed for their textural and fractal properties such as average cluster size, accessible surface area, and correlation dimension. The development of a 3D model such as this is a pivotal step toward computational optimization of porous materials for use in various applications, and being able to perform adsorption analysis of materials produced across a range of synthesis parameters would be crucial in advancing toward this reality, especially given that adsorption analysis is one of the most fundamental techniques used to characterize materials in experimental work. The work presented here, therefore, models adsorption analysis of the porous structures which have been created from our lattice-based Monte Carlo simulation, across varying catalyst concentrations and solids contents, the results of which can be directly compared to experimental analysis of RF gels which have been synthesized in the lab.

## 2 Methodology

### 2.1 Porous Structure Simulation Procedure

The structures analyzed in this work are produced from a three-dimensional lattice-based model, which simulates the formation and growth of porous materials through kinetic Monte Carlo cluster–cluster aggregation. A full description of the simulation process, in addition to the textural and fractal properties of the materials produced, can be found in the previously published works.^25,26^ The models presented both in our previous works and in this work were developed with the GNU Fortran compiler and GNU parallel tool.^27^

This simulation is performed on a 1 000 000 site lattice, where the desired solids content is achieved by populating a percentage of the lattice sites with monomers. The laboratory synthesis of RF gels includes a reaction between resorcinol and formaldehyde molecules with the addition of a basic catalyst, the presence of which leads to the formation of negatively charged resorcinol ions. These act as cluster seeds with which monomers can react, leading to the formation of monomer clusters. This process is modeled in the simulation by “activating” at random a percentage of the monomers on the lattice, with each activated monomer acting as a cluster seed for the simulation, and where the varying percentage of activated monomers is comparable to varying catalyst concentration (*C*_C_). In this research, activated monomer percentages of 0.1–4% are simulated, a range based on the proposed percentage of resorcinol molecules that are deprotonated by a basic catalyst during the RF reaction.^28^ Solids contents (*S*_C_) of 10–50% were used for the work presented here, once again selecting relevant values comparable to materials commonly synthesized experimentally.

**Figure 1:**
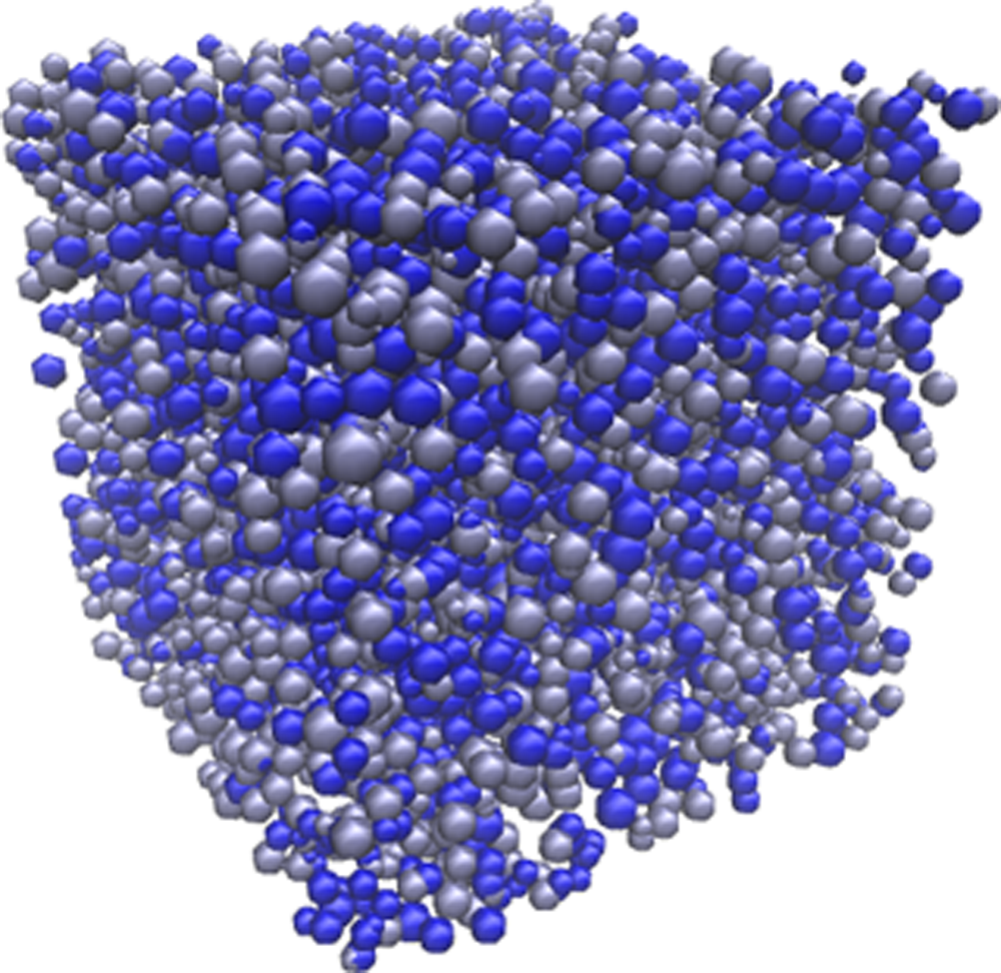
Simulated organic gel visualized in 3D at 30% solids content (*S*_C_) and 2% activated monomers (*C*_C_). Note that each sphere represents an individual cluster, and the different colors of clusters present are for visual purposes only.

Each simulation was repeated with 10 different seeds for the random number generator, resulting in 10 different structures at each *S*_C_ and *C*_C_ percentage. The final material is a porous network of primary spherical clusters similar to that of RF gels; however, the simulation could also be applicable to other porous materials whose formation mechanism operates under similar principles. Figure 1 shows the final simulated porous material, visualized in 3D, at 30% *S*_C_ and 2% *C*_C_.

### 2.2 Adsorption Analysis Procedure

Given that adsorption analysis is one of the most common methods employed to characterize porous materials experimentally, modeling this process computationally for simulated structures allows helpful comparisons to be drawn between experimental and computational results. The adsorption model used to analyze the cluster structure presented here is based upon work by Monson,^18^ which has been implemented in a three-dimensional lattice for this work so that it is applicable to individual pores as well as larger porous structures. This simulates the adsorption process using mean field theory for a lattice gas system, calculating the gas density across the lattice with varying relative activity—a chemical potential parameter relating directly to the relative pressure of the gas. A detailed explanation of the calculations carried out within this model is provided in the original paper by Monson.^18^

These calculations for adsorption analysis are based upon the mean field approximation of the system Helmholtz energy (*F*), shown in eq 1:
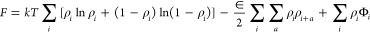
1where *k* is the Boltzmann constant, *T* is the system temperature, ρ_*i*_ is the average density of site *i* within the lattice, ∈ is the nearest neighbor interaction strength, and Φ_*i*_ is the external field at site *i*. The density distribution at equilibrium, where the overall density of the system is fixed, is related to chemical potential (μ) using eq 2:
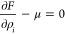
2

This relationship can then be used to calculate ρ_*i*_ at various μ values and subsequently calculate the average density of the system using the iterative procedure described in detail by Monson, assuming that μ is equal across the system. The system activity (λ) is then related to μ using eq 3:
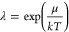
3

This is calculated for 2000 adsorption points and 2000 desorption points, and the resulting isotherms are plotted with respect to relative activity (*λ/λ*_0_), which is directly comparable to relative pressure (*p*/*p*_0_) in nitrogen adsorption experiments.^18^

Here, this model is used to simulate adsorption initially within individual 3D pores of varying widths and lengths, for both open transport pores and bottleneck pores which are closed at one end. This allows a comparison to be made between the simulated isotherms and those expected for the various pore sizes and geometries, therefore validating the model and providing a baseline for the isotherms generated from the simulated adsorption process within the porous structures. To simulate the adsorption process within the simulated porous material, the adsorption model was adapted to accommodate the 1 000 000 site lattice structure, and the final material from the cluster–cluster aggregation model was exported into the necessary format for the analysis to be carried out.

The adsorption analysis was carried out for 10 different simulated materials produced at each solids content and activated monomer percentage, and an average was taken across the 10 isotherms.

### 2.3 Adsorption Process Visualization

Given that the adsorption simulation calculates the density of each lattice site for various chemical potentials, the adsorption and desorption processes can be visualized as density profiles across the lattice at a given point on the isotherm. In this work, this is visualized as a vertical 2D slice through the center of the lattice—for both individual pores in addition to the simulated structures—and plotted as a color map using MATLAB. Here, the wall sites are visualized as a solid red color, while the pore sites are visualized using a color scale that is based upon the density value of each site, with higher density values indicating where adsorption has taken place. These density profiles provide insight into the visual differences between the adsorption and desorption processes and allow comparisons to be drawn between what is observed visually and what is observed within the isotherm.

### 2.4 Pore Size Analysis

The pore size distribution of the simulated structure can be analyzed using the isotherm data from the adsorption analysis, in the same manner as in experimental analysis. The method employed here is based on the Barrett, Joyner, and Halenda (BJH) theory,^29^ which is used frequently within experimental analysis, once again allowing more direct comparisons to be drawn between computational and experimental results. The BJH method is used to determine the pore size distribution and pore volumes within the meso- and macroporous range, assuming pores of cylindrical shape are present, with the principle of this method relying on the calculation of the Kelvin core radius of the pore at set pressure intervals using the desorption isotherm data.

The pore size calculations are predominantly based around the Kelvin core radius equation for desorption (eq 4), which defines the relationship between relative pressure (or, in this case, relative activity) and core radius:
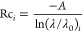
4where (λ/λ_0_)_*i*_ is the relative activity at point *i*, Rc_*i*_ is the corresponding Kelvin core radius at point *i*, and *A* is the adsorbate property factor—a value that accounts for properties such as surface tension and molar volume—and is equal to 0.953 for nitrogen gas.

When BJH analysis is carried out experimentally, an empirical formula is used to determine the thickness of the layer which remains adsorbed onto the pore walls after the core of the pore empties, the coefficients of the equation applying to interactions between specific adsorbents and adsorbates. The total pore radius can, therefore, be determined as the sum of the Kelvin core radius and the thickness of the adsorbed layer at each pressure interval, with pores of new core diameters emptying as desorption proceeds, and the thickness of the layer adsorbed onto the pore walls decreasing as further desorption takes place. A comparison of the total volume desorbed at each point on the isotherm to the corresponding adsorbed layer thickness indicates whether or not new pores are emptying as desorption takes place. In this computational work, however, the intervals within the 2000 desorption points on the isotherm are assumed to be small enough that the incremental desorption from the adsorbed layer will be negligible in comparison to the volume desorbing when a pore core empties.

The relationship between Rc_*i*_ and (λ/λ_0_)_*i*_ (eq 4) is, therefore, used to determine the diameter of the new pores that have been emptied, and the volume attributed to the emptying of these pores can then be used to plot pore size distributions for the structure being analyzed. In order to account for the layer remaining adsorbed onto pore walls after the core has been emptied, the calculated Rc_*i*_ is increased by a value of 1, estimating that a monolayer 1 site in thickness remains adsorbed. This analysis was carried out for the 10 isotherms produced through adsorption analysis for each solids content and activated monomer percentage, and an average was taken across the 10 resulting pore size distributions.

### 2.5 Gel Synthesis

To allow for accurate comparisons to be drawn between the simulated materials and those synthesized in the lab, the work here presents results from the experimental analysis of RF gels. A standard preparation method is carried out for each gel synthesis, the full details of which can be found within previous work carried out by the group.^30^

The RF gel synthesis involves the use of four reagents:

(1) resorcinol (SigmaAldrich, ReagentPlus, 99%);
(2) formaldehyde (as formalin solution, SigmaAldrich, 37 wt % formaldehyde in water and methanol);
(3) deionized water (produced in-house with Millipore Elix 5, Progard 2); and
(4) catalyst, sodium carbonate (SigmaAldrich, anhydrous, ≥99.5%).

These reagents were combined in separate glass containers according to the desired catalyst concentration, which is generally quantified with respect to the resorcinol/catalyst molar ratio (*R*/*C* ratio). All gels were prepared at a solids percentage of 20%, and a resorcinol/formaldehyde molar ratio of 1:2 in accordance with the stoichiometry of the accepted RF reaction.^31^ The containers were sealed and placed in a Memmert ULE-500 oven at a temperature of 85 °C for 3 days, where the gelation process took place. Following gelation, a 3 day solvent exchange procedure was performed, where the water within the porous hydrogel was replaced by acetone—a solvent possessing a significantly lower surface tension value—which is a necessary step to reduce the extent of structural collapse during drying. All gels were dried at 85 °C for 2 days using a vacuum oven (Townson and Mercer 1425 digital vacuum oven) with an attached vacuum pump, resulting in the formation of final materials known as xerogels.

### 2.6 Experimental Nitrogen Adsorption Analysis

As with the synthesis process of the RF gels, the nitrogen adsorption method used here follows a standard procedure which has been documented in detail within previous work carried out by the research group.^30^ A dried gel sample of approximately 0.5 g was degassed before undergoing the nitrogen adsorption analysis using a Micromeritics ASAP 2420 surface area and porosity analyzer. The adsorption analysis lasts around 20–30 h per sample, collecting 40 data points for adsorption as the relative pressure is incrementally increased from 0.1 to 1 and then 30 data points for desorption as the relative pressure is decreased from 1 to 0.1. The Micromeritics ASAP 2420 equipment software is utilized to analyze the isotherm data to provide results such as the sample’s BJH pore size distribution, total pore volume, accessible surface area, and average pore width.

## 3 Results and Discussion

### 3.1 Computational Adsorption Isotherms

**Figure 2:**
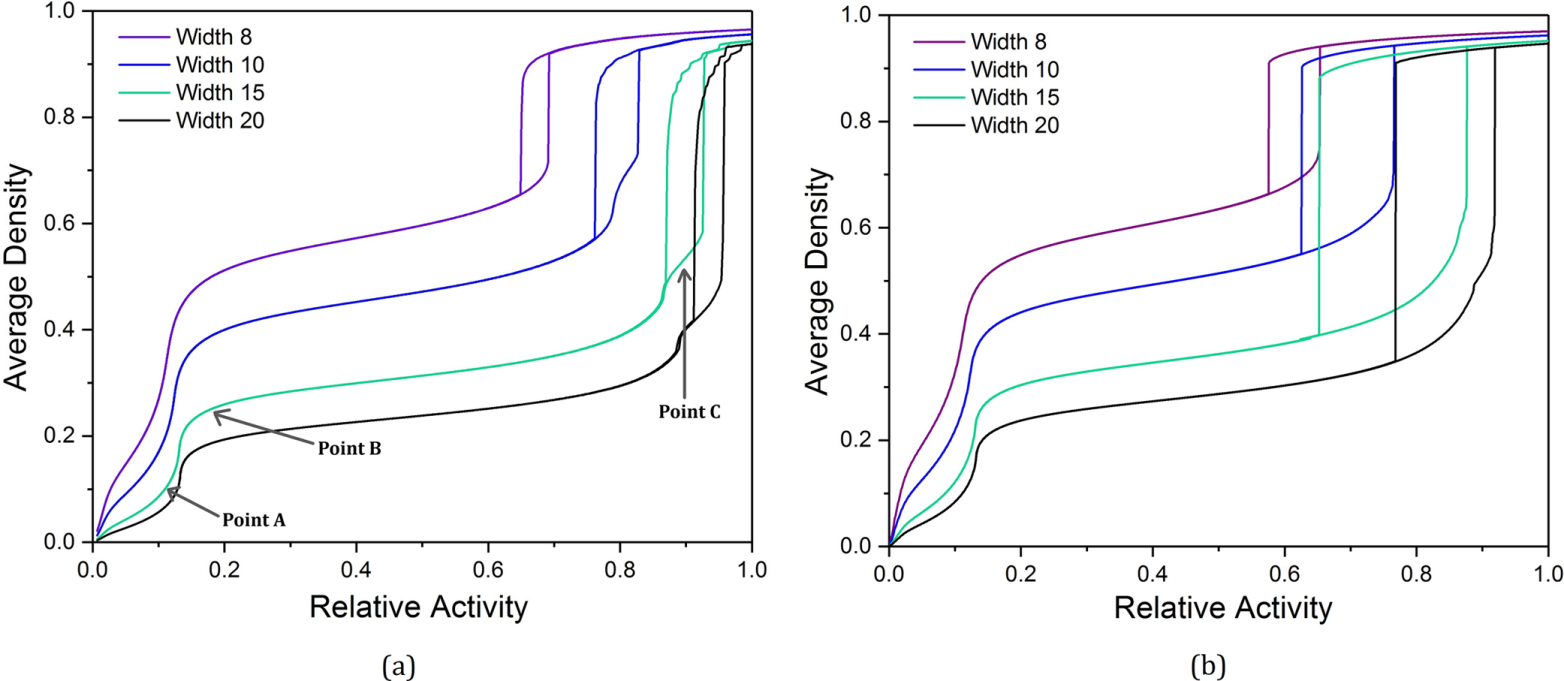
Simulated isotherms for adsorption and desorption within individual pores of varying widths in (a) open transport pores and (b) bottleneck pores.

Figure 2a displays the simulated isotherms from adsorption analysis of open rectilinear transport pores with varying pore widths, all of which are measured in terms of lattice sites and possess pore lengths of 40 sites. Figure 2b shows simulated isotherms of bottleneck pores, also possessing a length of 40 sites, with a bottleneck entrance width approximately one-third of the total pore width, rounded to the nearest integer. As discussed, previous experimental and computational works have demonstrated the significant effect pore width has on adsorption behavior and therefore on the shape of the isotherm produced, and this is reflected in the work presented here. Across both sets of isotherms, as the pore width is increased, the adsorption uptake is more gradual, and the hysteresis loop—the point at which the pore fills and empties—shifts to higher values of relative activity on the *x*-axis. Furthermore, the shape of the isotherms within Figure 2a agrees with that of pores open at both ends found in previous studies, where a relatively narrow hysteresis with two largely parallel lines is observed. The wider, more gradual hysteresis loops observed within Figure 2b, meanwhile, are in agreement with those observed in previous studies for bottleneck pores.

It is possible to identify two or three distinct sections of these isotherms, depending on the width of the pore. First, there is an initial rapid uptake at **Point A** on the isotherm, which then begins to plateau at **Point B**. For larger pore widths, **Point C** can be observed—a second point at which a rapid uptake begins to plateau—before pore filling takes place. Each of these points will be discussed in Section 3.2 where their corresponding density profiles will be analyzed.

### 3.2 Adsorption Density Profiles

**Figure 3:**
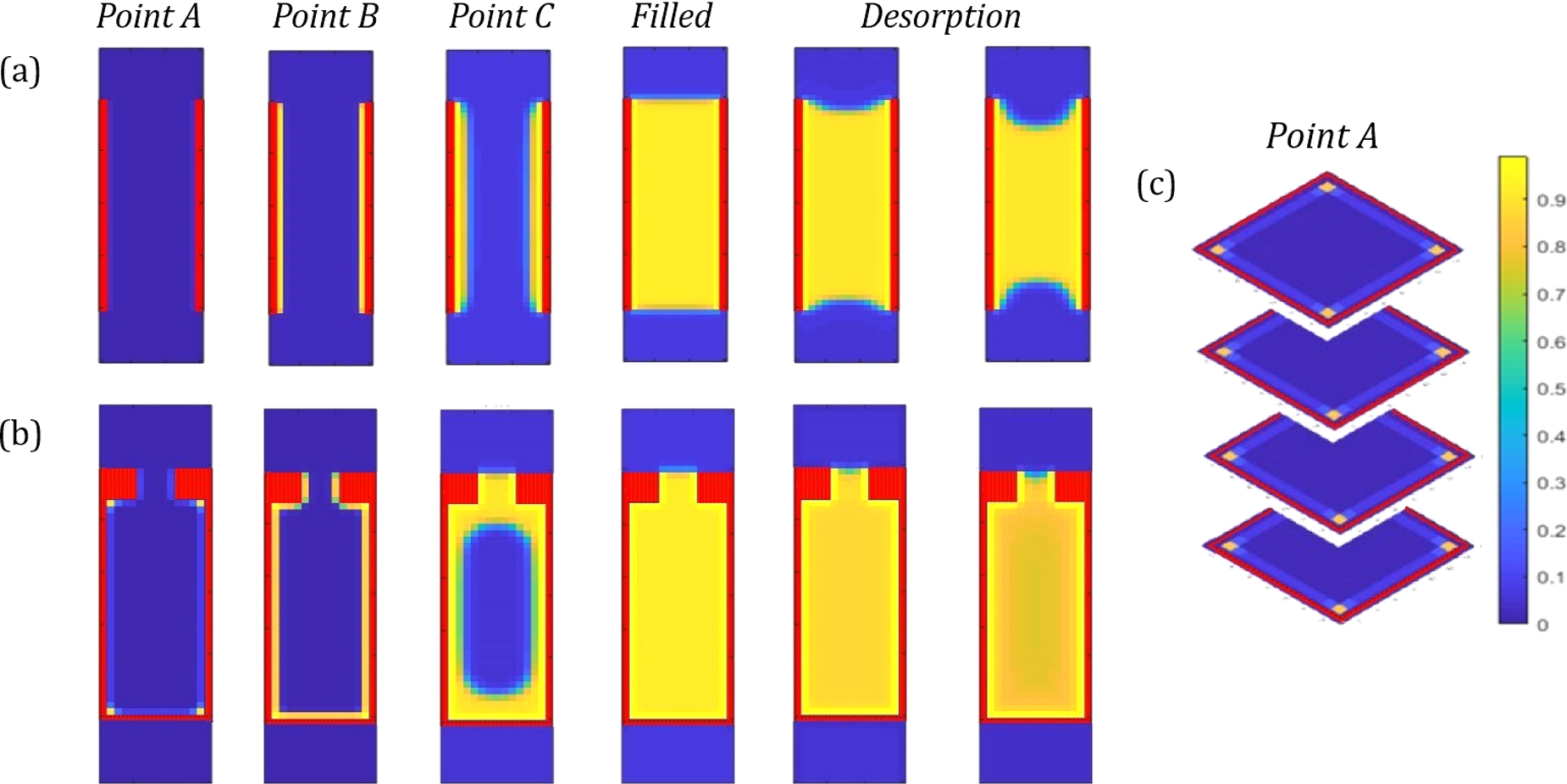
Vertical density profiles of the center of the pore at various stages in the adsorption and desorption process for open transport pores (a) and bottleneck pores (b), in addition to horizontal density profiles for an open pore showing initial adsorption along the corners within the pore (c). Red sites show pore wall sites, blue sites empty sites within the pore, and yellow sites where adsorption has taken place, in accordance with the density color scale shown.

In order to further investigate the adsorption process taking place at each of the points identified, in addition to other significant points within the isotherm, the pore density distribution data can be used to produce density color maps. These show, pictorially, where adsorption has taken place within the pore at each of the stages selected, further elucidating the mechanism by which adsorption and desorption occur. Figure 3a shows color map density profiles of the pore at the different adsorption points indicated for an open transport pore 15 sites in width, displaying a two-dimensional slice down the center of the pore, where the sites with red markers correspond to pore wall sites, and the color of the density profile transforms from blue to yellow as adsorption occurs. Upon inspection of the density profile at **Point B** within Figure 3a, it is clear that the plateau observed on the isotherm here corresponds to the formation of a monolayer across each of the four pore walls, after which point further adsorption takes place gradually. In pores of sufficient width, we observe the feature at **Point C**, which corresponds to the formation of a second layer of adsorbed gas on top of the original monolayer, as depicted on the corresponding density profile, just before the pore fills. The remaining density profiles display the pore as desorption takes place—beginning from the filled state at a relative activity of 1 and then showing the initial desorption as relative activity decreases, and finally the pore just before emptying at the desorption branch of the hysteresis. The density profiles show that, while the pore fills through the gradual adsorption along the length of the pore walls, it conversely empties through the gradual removal of layers from the meniscus, revealing the difference in the mechanism by which adsorption and desorption takes place.

The adsorption and desorption process in bottleneck pores is also shown, once again using the central vertical density profiles, displayed in Figure 3b. Similar to the adsorption process taking place in open transport pores, monolayer formation is observed along the walls of the pore at lower relative activities, proceeded by multilayer formation and saturation of the bottleneck entrance area before the pore itself fills completely. Desorption, once again, takes place through the removal of layers from the meniscus at the bottleneck entrance, before the pore itself empties completely when a sufficiently low relative activity is reached. The difference in mechanisms for adsorption and desorption is more prominent for bottleneck pores as the pore filling takes place much more gradually through the narrow entrance, in contrast to the more immediate emptying of the pore at lower relative activities. This is in agreement with the IUPAC hysteresis classifications where the H2 hysteresis loops, which are associated with materials composed of bottleneck pores, indicate the gradual filling of pores during adsorption and the sudden emptying of pores during desorption.

Despite showing adsorption taking place on the isotherm at **Point A**, the corresponding density profile for the 2D central slice of the open transport pore at this stage appears to show no adsorption taking place. Given that the model is in 3D, horizontal density profiles of the cross-section along the length of the pore can be produced, as shown in Figure 3c, allowing areas of the pore not detected by the central vertical profile to be analyzed. Here, the cross-section of the pore at **Point A** on the isotherm is illustrated, where we can see adsorption taking place at the corner sites along the length of the pore where two walls meet, which explains this initial adsorption failing to appear on the central vertical density profile. This initial adsorption along the pore corners was also observed for bottleneck pores, this time including adsorption onto the corners of the pore walls below the bottleneck entrance.

Modeling adsorption within individual pores in this way is valuable, not only in confirming the theories behind the analysis of our laboratory experimental work but also in providing additional insight into specific mechanisms, such as the pore filling taking place along the walls and emptying via the meniscus. Furthermore, given that the results presented for individual pores from this model are in agreement with those cited in the literature, the utility of the adsorption calculations is verified before being applied to the 3D complex structures generated from the kinetic Monte Carlo cluster aggregation simulation.

### 3.3 Adsorption in Porous Structures

#### Varying Solids Content

**Figure 4:**
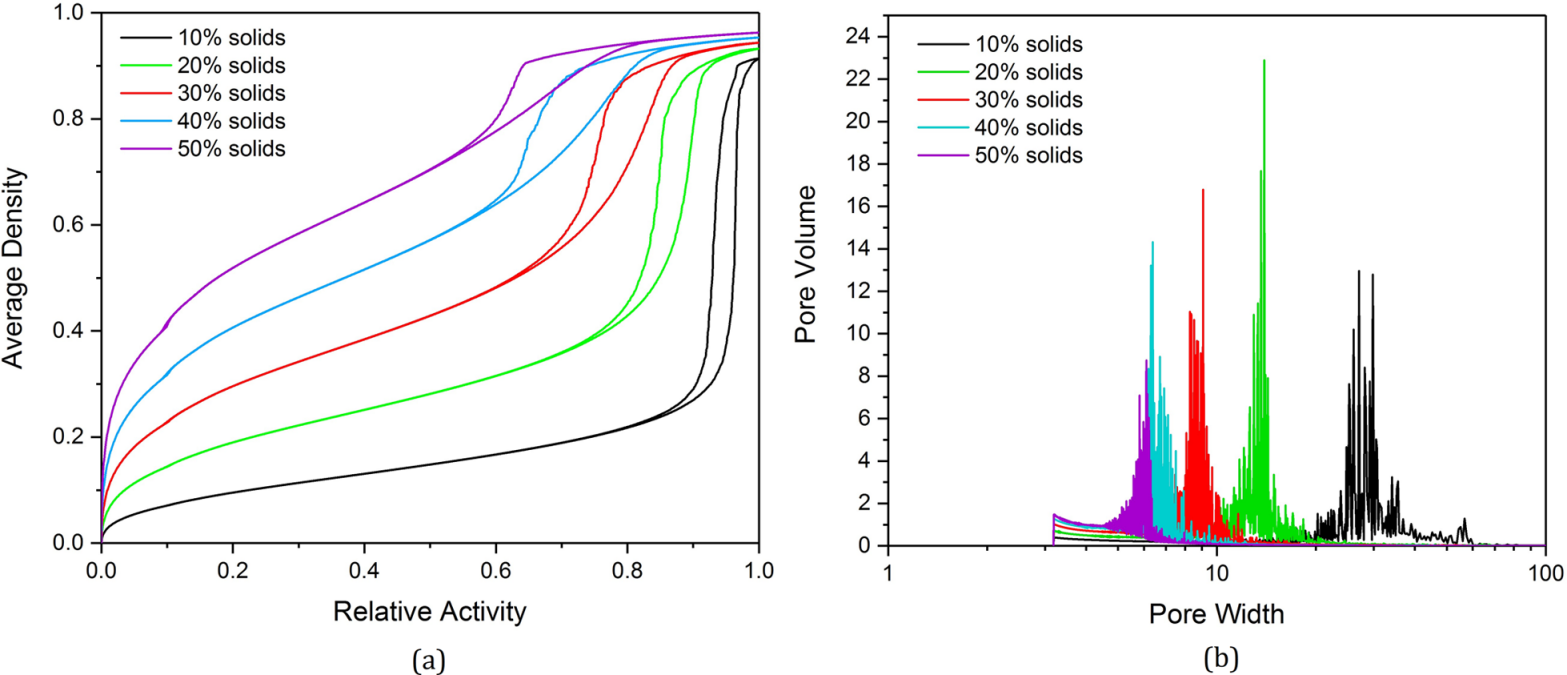
(a) Simulated isotherms for the adsorption analysis of model porous structures at 1% activated monomers (*C*_C_) and varying solids contents (*S*_C_). (b) Corresponding pore size distributions.

Figure 4a shows the resulting isotherms from adsorption analysis of the porous structures produced from the kinetic Monte Carlo cluster aggregation model at 1% activated monomers (*C*_C_) with varying solids contents (*S*_C_). We can observe the changes in hysteresis loop shape across the varying *S*_C_ percentages, with structures at lower *S*_C_ producing isotherms with narrow, elongated hysteresis loops, indicating the presence of open transport pores. This is in contrast with those at higher *S*_C_, which possess wider, shorter hysteresis loops, indicating the presence of bottleneck pores within the structure. The changes in the *x*-axis position of the hysteresis loop can also be observed, shifting from high to low relative activity values as the solids content is increased from 10% to 50%, indicating that higher solids contents result in structures with narrower pores, as demonstrated within the results already presented in Section 3.1 on varying pore widths. Figure 4b shows the pore size distribution results from the subsequent BJH pore size analysis of structures at varying solids contents of 10–50%, where the calculated pore size is measured in lattice sites. These distributions agree with the visual analysis of the adsorption isotherms—quantifying the shift in pore size as solids content is altered, where pores become narrower as the structures become more densely packed with higher solids contents. The distribution of pore width also narrows with increasing solids content, where structures at lower solids percentages possess a wider range of pore sizes in comparison to those at higher solids percentages. An increase in volume is observed toward the lowest pore widths (between width values of approximately 3–5 sites) for each distribution in Figure 4b—this can be attributed to the final layers of adsorbed gas remaining on pore walls, which are the last to desorb from the structure, and the total volume of which will increase for structures of higher solids contents as a result of the increased surface area available.

#### Varying Catalyst Concentration

**Figure 5:**
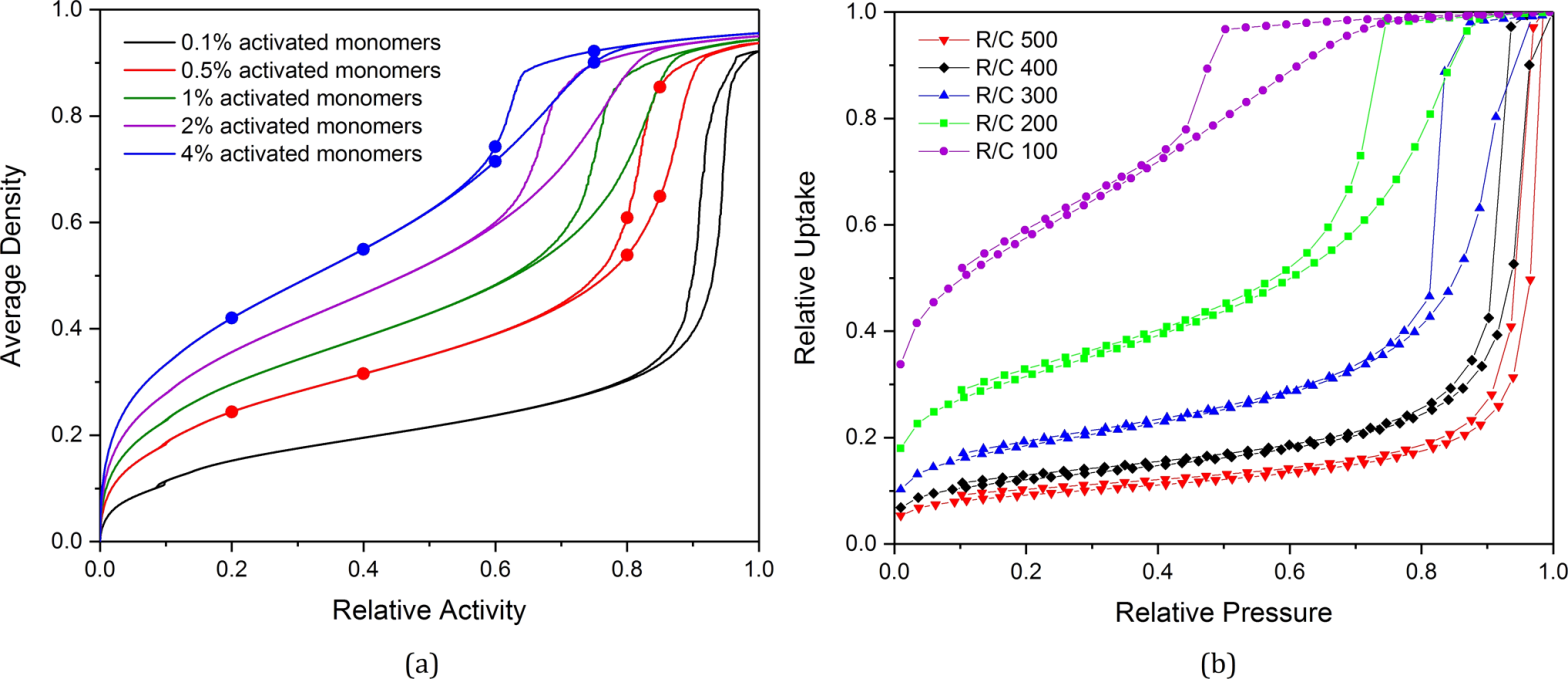
(a) Simulated isotherms for the adsorption analysis of model porous structures at 30% solids content (*S*_C_) and varying catalyst concentrations (*C*_C_). (b) Experimental isotherms for the adsorption analysis of RF xerogels synthesized in the lab at varying catalyst concentrations (*R*/*C* ratios), where low *R*/*C* ratios correspond to higher catalyst concentrations. Note that the circular points indicated on simulated isotherms in Figure 5a correspond to those visualized in Figure 7.

The adsorption process was also simulated across structures with varying *C*_C_ values, the isotherms for which are shown in Figure 5a at 30% *S*_C_, with the results indicating similar trends to those observed for varying *S*_C_. Once again, the position of the hysteresis loop on the *x*-axis shifts toward lower relative activity, pointing toward the presence of pores that are narrower in width. The changing appearance of the hysteresis loop from narrow and elongated in shape to wider and shorter points toward the changing geometry of the pores themselves, with lower *C*_C_ structures comprising open transport pores and higher *C*_C_ structures comprising bottleneck pores.

**Figure 6:**
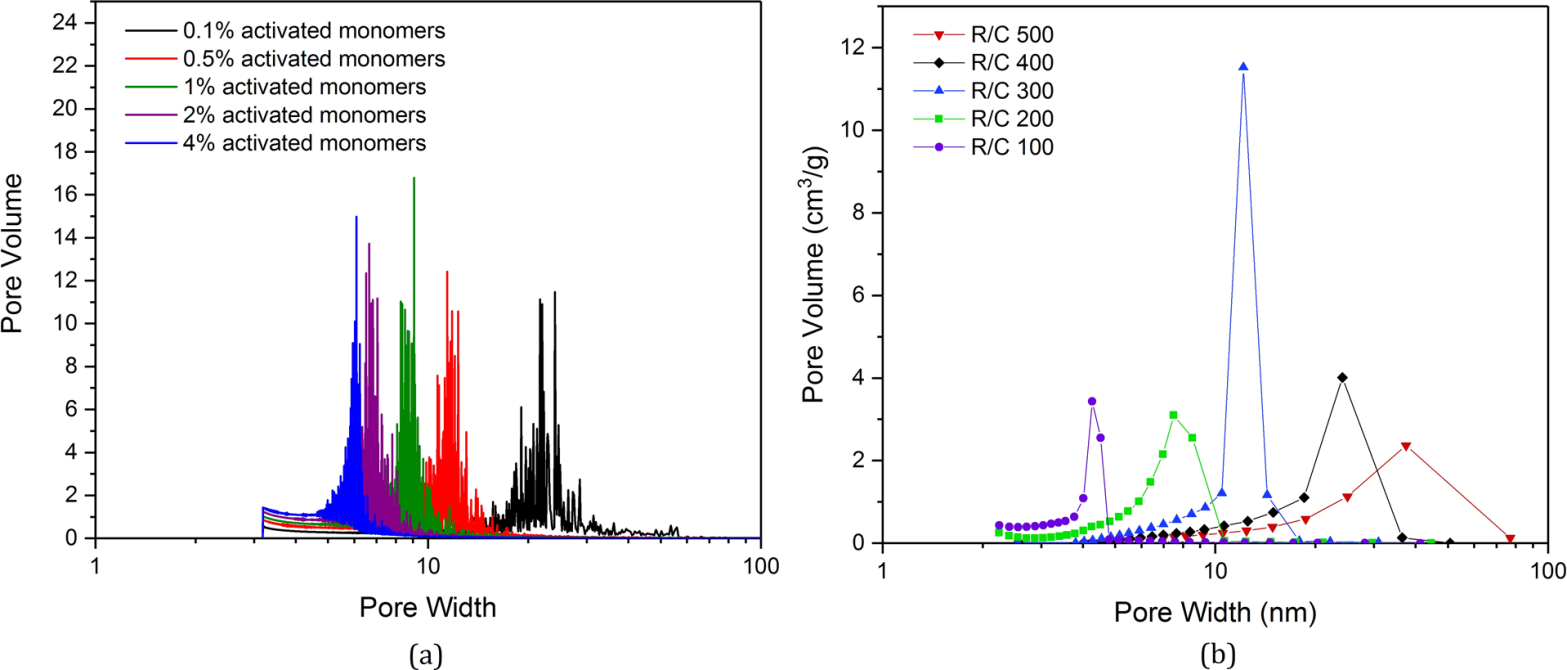
(a) Pore size distributions of model porous structures at varying catalyst concentration (*C*_C_). (b) Pore size distributions for RF xerogels synthesized in the lab at varying catalyst concentrations (*R*/*C* ratios), where low *R*/*C* ratios correspond to higher catalyst concentrations.

These simulated adsorption isotherms can also be directly compared to those obtained experimentally through nitrogen adsorption experiments for RF gels synthesized in the lab, shown in Figure 5b, for RF gels at varying catalyst concentrations, where high resorcinol/catalyst (*R*/*C*) ratios correspond to low catalyst concentrations, and low *R*/*C* ratios correspond to high catalyst concentrations. Experimental isotherms have been plotted based on their relative uptake, allowing them to be compared directly to those from the adsorption analysis of model structures. Note that the simulated isotherms are shown for structures at 30% solids content, while the experimental isotherms are shown for RF gels synthesized at 20% solids content. This comparison is made because the laboratory-synthesized gels are subject to shrinkage during drying, making their final solids content more comparable to the higher simulation values. Simulating shrinkage of the simulated materials produced from the kinetic Monte Carlo model and comparing these to the dried RF gels synthesized in the lab, although not performed here, would be useful for future work. The visual similarities between the experimental and simulated isotherms across varying catalyst concentrations are significant, with the same trends observed in the shape and position of the hysteresis loop. These trends are reflected once again in the pore size distribution results from the BJH analysis, both simulated (Figure 6a) and experimental (Figure 6b), displaying the shift from wider pores at lower catalyst concentrations to narrower pores at higher catalyst concentrations. These comparative results are valuable not only for the validation of the kinetic Monte Carlo cluster aggregation model for the formation of RF gels but also in showing promise in the potential for computational tailoring of these materials to optimize their performance in various applications.

### 3.4 Adsorption Process Visualized

**Figure 7:**
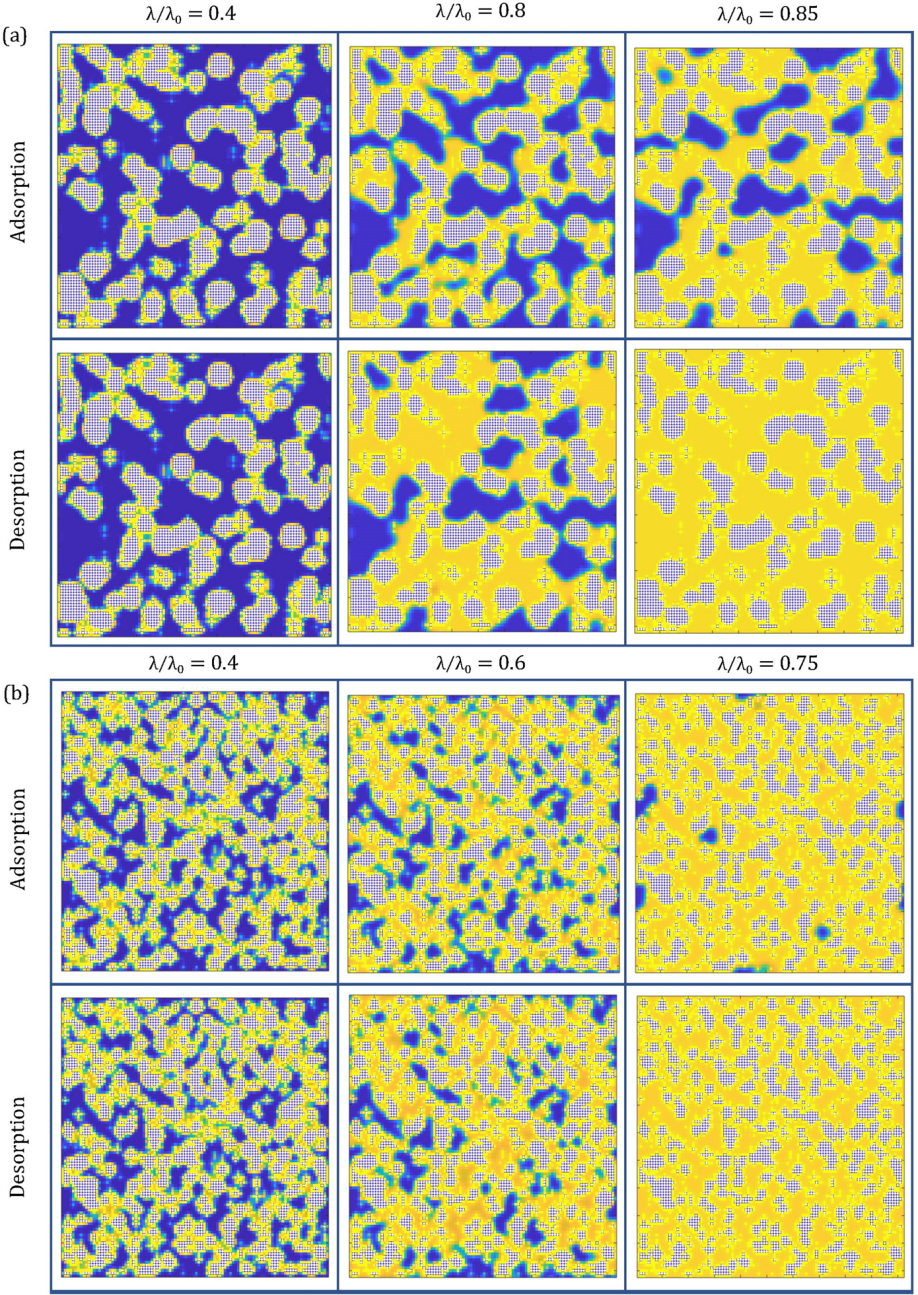
Density profiles through the center of the model porous structure at various relative activity (λ/λ_0_) values throughout the adsorption and desorption processes for (a) 30% solids content (*S*_C_) and 0.5% activated monomers (*C*_C_), and (b) 30% *S*_C_ and 4% *C*_C_. White sites show the material structure, blue sites empty sites within the pores, and yellow sites where adsorption has taken place.

In addition to the isotherm data produced from the simulated adsorption analysis, density profiles across the porous structures were generated at each point throughout the adsorption and desorption process. This is a useful way to visualize processes that cannot be observed by eye in experimental analysis. Figure 7a,b shows the visualized adsorption and desorption processes within structures produced at 0.5% and 4% *C*_C_, respectively, both at 30% *S*_C_. The density profiles across the structures are shown at the same relative activity values on the adsorption and desorption branches of the isotherm, corresponding to the markers located on the plots within Figure 5a and showing the visual differences between each. This provides a visual comparison between the mechanism by which pores fill during adsorption and empty during desorption. The differences are particularly evident in the 0.5% *C*_C_ structures, as shown by Figure 7a, where at a relative activity value of 0.85, the desorption branch shows a completely saturated structure while the adsorption branch shows many pores still yet to be filled. This highlights the differing mechanisms by which complex structures adsorb and desorb gases, which could have significant implications when it comes to the use of these materials in various applications. These images have also been compiled in video files, showing the adsorption and desorption processes at different points within the isotherm, which are available within the Supporting Information.

## 4 Conclusions

To conclude, the adsorption model presented here successfully captures the adsorption and desorption processes that take place both within individual pores and within complex porous structures, offering a computationally efficient method of simulating and analyzing materials such as RF gels, in contrast with the computationally expensive models which have been employed in previous studies.

The impacts of varying the width and geometry of individual pores were explored through analysis of the isotherm data produced, the results of which are in agreement with those found in the literature from both experimental and computational methods. The effect of varying solids content and catalyst concentration on the adsorption and desorption behavior of the porous structures was also assessed, as demonstrated by the changes in isotherm shape in addition to the visual differences observed from the density profiles at varying relative activities. The changes observed within the isotherm plots provided insight into the size and geometry of the pores present within the materials, with structures produced with lower solids content and catalyst concentrations comprising open transport pores which are larger in width, while those at higher solids content and catalyst concentrations comprised bottleneck pores which were narrower in width. The adsorption and desorption processes were visualized using density color maps, providing a visual comparison between the mechanism by which porous structures fill and empty—an imperative consideration when assessing the structural characteristics required for specific applications.

The results of this study also further validate the kinetic Monte Carlo cluster aggregation model from our previous works in capturing the formation of porous materials such as RF gels, as the simulated adsorption analysis results show significant similarities to those obtained experimentally for RF gels synthesized in the lab. The trends observed in the shape of the isotherm and position of the hysteresis loops are consistent between the two, as are the trends observed from the two BJH pore size distributions.

Overall, the results presented here show significant promise in advancing toward the computational tailoring of materials such as these in a manner that is realistically applicable to widespread industry use. A model that can predict and control a material’s properties in this way would be invaluable to realizing its full application potential, allowing determination of the synthesis parameters required to produce materials with the desired characteristics in a time-efficient and computationally inexpensive manner.

## Supporting Information Available

Video of the adsorption process for structures at 30% *S*_C_ and 0.5% *C*_C_ (MP4)
Video of the adsorption process for structures at 30% *S*_C_ and 4% *C*_C_ (MP4)
